# DIPS (Dystonia Image-based Programming of Stimulation: a prospective, randomized, double-blind crossover trial)

**DOI:** 10.1186/s42466-021-00165-6

**Published:** 2021-12-20

**Authors:** Florian Lange, Jonas Roothans, Tim Wichmann, Götz Gelbrich, Christoph Röser, Jens Volkmann, Martin Reich

**Affiliations:** 1 Department of Neurology, University Hospital and Julius Maximilian University, Josef-Schneider-Straße 11, 97080 Würzburg, Germany; 2 Institute for Clinical Epidemiology and Biometry (ICE-B) at the University of Würzburg, Josef-Schneider-Straße 2, 97080 Würzburg, Germany; 3 Clinical Trial Center (CTC) at the University of Würzburg, Josef-Schneider-Straße 2, 97080 Würzburg, Germany

**Keywords:** Deep brain stimulation, Dystonia, Imaging-guided DBS programming

## Abstract

**Introduction:**

Deep brain stimulation of the internal globus pallidus is an effective treatment for dystonia. However, there is a large variability in clinical outcome with up to 25% non-responders even in highly selected primary dystonia patients. In a large cohort of patients we recently demonstrated that the variable clinical outcomes of pallidal DBS for dystonia may result to a large degree by the exact location and stimulation volume within the pallidal region. Here we test a novel approach of programing based on these insights: we first defined probabilistic maps of anti-dystonic effects by aggregating individual electrode locations and volumes of tissue activated of > 80 patients collected in a multicentre effort. We subsequently modified the algorithms to be able to test all possible stimulation settings of de novo patients in silico based on the expected clinical outcome and thus potentially predict the best possible stimulation parameters for the individual patients.

**Methods:**

Within the framework of a BMBF-funded study, this concept of a computer-based prediction of optimal stimulation parameters for patients with dystonia will be tested in a randomized, controlled crossover study. The main parameter for clinical efficacy and primary endpoint is based on the blinded physician rating of dystonia severity reflected by Clinical Dystonia Rating Scales for both interventions (best clinical settings and model predicted settings) after 4 weeks of continuous stimulation. The primary endpoint is defined as “successful treatment with model predicted settings” (yes or no). The value is “yes” if the motor symptoms with model predicted settings are equal or better (tolerance 5% of absolute difference in percentages) to clinical settings. Secondary endpoints will include measures of quality of life, calculated energy consumption of the neurostimulation system and physician time for programming.

**Perspective:**

We envision, that computer-guided deep brain stimulation programming in silico might provide optimal stimulation settings for patients with dystonia without the burden of months of programming sessions. The study protocol is designed to evaluate which programming method is more effective in controlling motor symptom severity and improving quality of life in dystonia (best clinical settings and model predicted settings).

*Trial registration* Registered with ClinicalTrials.gov on Oct 27, 2021 (NCT05097001).

## Introduction

Dystonia is a central nervous system disease associated with sustained or intermittent muscle contractions causing abnormal, often repetitive, movements, postures, or both [1]. Activities of daily living, social participation and quality of life can be markedly impaired in dystonia due to pain, reduced overall mobility and stigmatization [2, 3].

Deep brain stimulation (DBS) is a surgical therapy for a variety of neurological and psychiatric disorders that uses continuous electrical stimulation of deep brain nuclei to alter neural activity. There is class I evidence for the safety and efficacy of DBS applied to the internal globus pallidus (GPi) to suppress motor symptoms in dystonia [4, 5] with an average improvement of dystonia severity of 50 to 60% in most clinical studies [5– 8]. However, there is a large variability in clinical outcome with up to 25% non-responders (< 25% motor score improvement) even in highly selected primary dystonia patients. In a large cohort of patients collected from several European centers we recently demonstrated that the variable clinical outcomes of pallidal DBS for dystonia may result to a relevant degree by the exact location and stimulation volume within the pallidal region [9].

These results and the underlying cohort of > 80 patients were subsequently used to derive probabilistic maps of anti-dystonic effects by aggregating individual electrode locations and volumes of tissue activated (VTA). These maps and novel computer algorithms showed robustness between the predicted and observed clinical improvement with an R^2^ of 0.53 (*p* < 0.01) in a leave-one-out-cross-validation. On average, individual predictions differed from observed improvements in dystonia by only 16.9 ± 11.6%. Interestingly, the computer algorithms predicted a potential improvement for the cohort and a reduction in non-responders if patients were switched to the optimal stimulation settings determined by the algorithms (mean improvement: 16.9 ± 11.6% and 25% to < 5% non-responders) [9].

We subsequently modified the algorithms to test all possible stimulation settings of de novo patients in silico based on the expected clinical outcome and thus potentially predict the best possible stimulation parameters for the individual patients. Within the framework of a BMBF-funded study, this concept of a computer-based prediction of optimal stimulation parameters for patients with dystonia will be tested in a randomized, controlled crossover study.

## Methods

### Aim of the trial

Deep brain stimulation for dystonia is in principle highly effective, but clinical application lacks standardization and the therapy is largely skill and experience based, which results in variable patient outcomes, even in experienced centers. Prolonged latencies between changes in program settings and observed symptom changes (sometimes weeks or months) hinder optimization of stimulation settings based on clinical response evaluation, which is the gold-standard for parameter titration. The parameter space of DBS is confusingly large and the high numbers of variables and resulting stimulation options (> 1.000.000 options with modern DBS systems) prevents an exhaustive clinical search for the best possible settings. We envision, that this search could be reliably performed by our digital expert system, which would provide one or a few programming suggestions, which could then be further refined in clinical practice. Such an approach would be advantageous to patients and physicians, by reducing the programming burden, providing faster and more efficient symptom control for patients and reduced programming g time for neurologists.

### Study description and study design

**Fig. 1:**
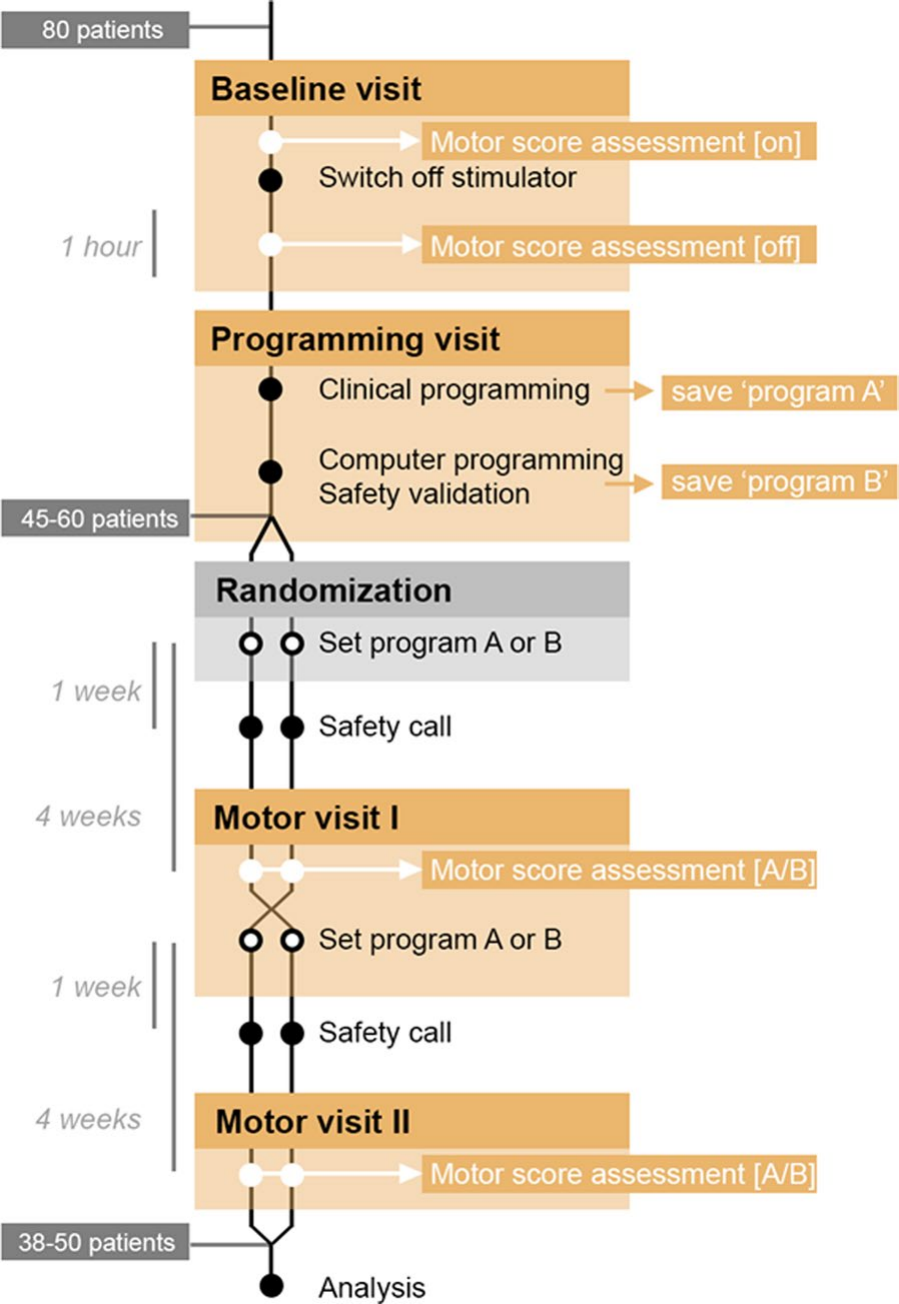
provides an overview of the course of study. Patients first undergo baseline visit and programming visit, then are randomized and blinded to two arms and receive either the computer-generated or the clinically generated program for 4 weeks. In the first motor visit, the patients are examined and then—still blinded—receive the other program for four weeks. Subsequently, in the second motor visit, the program is evaluated and the patients end the study with the choice of the better program

This study will be conducted as a double-blind, randomized, cross-over trial. The overarching study flow is illustrated by Fig. 1. The study includes five phases: baseline visit, programming visit, randomization, motor visit I, and motor visit II.

**Fig. 2:**
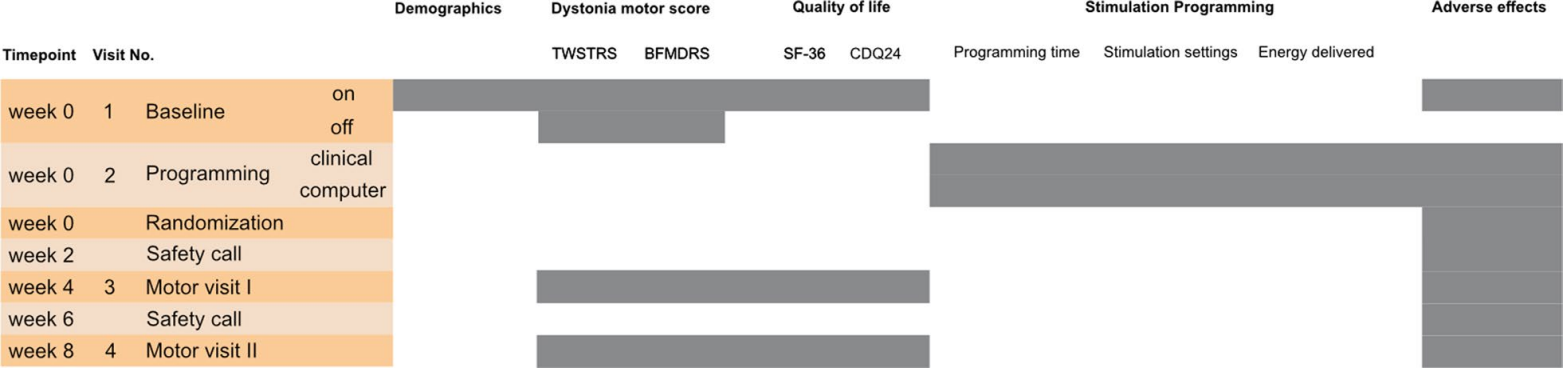
gives an overview of the examinations per visit. The visits are represented as a row below each other, the respective examinations as marked columns on the right-hand side

*At baseline visit*, standardized recordings of medical history and motor examination are performed. In addition to the neurological examination, the motor examination includes the video recording of the internationally used dystonia motor scores Toronto Western Spasmodic Torticollis Rating Scale (TWSTRS) and Burke–Fahn–Marsden Dystonia Rating Scale (BFMDRS). Health-related quality of life is assessed using SF-36 and CDQ24. To determine the immediate stimulator effect, the stimulator is switched off and the patients are examined in this Stim OFF condition (TWSTRS and BFMDRS) after 60 min. For a graphical representation of the visits and the respective examinations, please see Fig. 2.

During *programming visit*, a DBS expert performs comprehensive reprogramming according to clinical best practices. This represents the usual procedure for initial setting of the stimulator after surgery. This process is described in detail in the section Clinical programming below.

According to *randomization* the unblinded programmer will activate one of the two programs (“*computer” or* “*clinical”*). Adequate measures to keep the patient blinded to the respective settings will be taken (ramp settings > 8 s and patient programmer display reduced to minimal settings). Patients will stay under physician supervision for around 30 min to check for any acute side effects.

*Motor visit 1* is scheduled 4 (± 1) weeks after randomization as the first follow-up. Again, standardized video recordings of the motor examination (TWSTRS/BFMDRS) and patient questionnaires (SF-36, CDQ24) will be taken to evaluate symptom severity and disease-related quality of life. The unblinded DBS programmer will then swap the programs (“*computer” to* “*clinical or* “*clinical” to* “*computer”*). Again, patients will stay under physician supervision for around 30 min to check for any acute side effects and will then be released into the second chronic phase. A telephone safety call 1 week after each change of stimulation parameters was implemented to allow for early response to any problems or suboptimal motor control.

*Motor visit 2* is scheduled 8 (± 1) weeks after randomization as the second follow-up. A third time, standardized video recordings (TWSTRS/BFMDRS) and patient questionnaires (SF-36, CGI) will be taken to evaluate symptom severity and patient’s program preference will be evaluated. The study concludes and the patients can decide which program will be activated for chronic use.

### Arms and interventions

#### Image-based prediction of stimulation settings

In the preparation of the *programming visit,* the preoperative MRI to localize deep brain nuclei and the postoperative CT to localize electrode placement will be analyzed by the computer algorithm. The software will then propose individual stimulation settings that yield high in silico potential for optimal antidystonic effect. Addressing efficacy and safety concerns we opted to define upper and lower boundaries for the amplitude range, which were derived in a data driven approach by using two standard deviations around the mean amplitude of the original cohort (mean = 3.4 mA, lower limit: 1.5 mA, upper limit: 5.5 mA) [10]. Having limits of 1.5 and 5.5 mA and a step size of 0.5 mA, there are 9 possible stimulation settings for each contact. The algorithm will check 4 heights representing 4 contacts, in case of directional electrode systems, these directional contacts will be used in ring-mode. This gives 4 × 9 for each side raised to the power of 2 for both hemispheres: 1296 possible combinations in total for each patient. These stimulator settings are than sorted by efficacy and filtered in a two step-manner. Firstly, the settings are removed if they share less than 50% overlap with the underlying model to maintain high confidence in the corresponding clinical efficacy. Secondly, outliers in the stimulation amplitude are excluded if the amplitude was one standard deviation above the average of the top 5 predictions. The proposed settings are checked for plausibility and safety by a DBS expert and saved on the stimulator as "computer"-program. For each stimulation side, one alternative stimulation setting will be proposed, in case the first option leads to otherwise not manageable side effects.

#### Clinical programming

The clinical programming will be performed by comprehensive reprogramming according to clinical best practices and represents the usual procedure for initial setting of the stimulator after surgery. This method and its exact algorithm have been published by Steigerwald and colleagues [11], and will only be briefly summarized here: the IPG is set as anode (+) and stimulation frequency and pulse width kept constant at 130 Hz and 120 μs, respectively. Than, each electrode is stimulated as monopolar cathode (−) by increasing the amplitude to a maximum of 6 V, as long as no adverse effects are elicited. The contact with the most beneficial effect is chosen for long-term stimulation. If no contact with acute beneficial effect can be determined, a contact that elicits phosphenes at an amplitude above 3 V was selected. If phosphenes are induced below 3 V, the next proximal electrode is chosen. The amplitude will be set to 0.5 V below the threshold of eliciting adverse. The resulting program is saved on the stimulator as "clinical"-program.

### Outcome measures

The primary efficacy endpoint is based on the blinded physician rating of dystonia severity reflected by Clinical Dystonia Rating Scales (BFMDRS or TWSTRS). The raters will be internationally recognized leading experts in the field of movement disorders that are not part of the study team or otherwise related to our center. For both interventions (best clinical settings and model predicted settings) we will calculate the symptom severity score after 4 weeks of continuous stimulation, expressed in percent of the pre-operative score. The primary endpoint is defined as “successful treatment with model predicted settings” (yes or no). The value is “yes” if the motor symptoms with model predicted settings are equal or better (tolerance 5% of absolute difference in percentages) to clinical settings.

Secondary endpoints will include measures of quality of life (SF-36 and CDQ24 questionnaire), calculated energy consumption of the neurostimulation system and physician time for programming. The study aims at estimating the success rate and explorative characterization of treatment failures in preparation for a confirmatory multicentre clinical trial.

#### Power calculation/sample size

The main conclusion from the success rate will be: (1) If the success rate is 80% or better, plan a superiority trial; (2) if the success rate is about 50%, plan an equivalence trial or, alternatively, examine the combination of best clinical practice and machine-based advice; (3) if the success rate is 20% or below, further exploratory work and considering other mathematical techniques is supported rather than a larger clinical trial. The data should be able to discriminate between these three scenarios in the sense that at most one of these three scenarios is in the 95% CI for the empirical success rates. This will be achieved with a sample size of n = 40 evaluable participants. A number of successes of 27 or more/14 to 26/13 or less does then rule out success rates of less than 50% / more than 80% or less than 20%/more than 50%, respectively, in the sense of the 95% CI.

We plan a data driven stopping if (1) 27 successes have occurred OR (2) at least 14 but no more than 26–k successes have occurred in 40–k patients for some number k = 1…12 OR (3) no more than 13–m successes have occurred in 40–m patients for some number m = 1…13, because it is then clear which two of the three rates 80%, 50% and 20% would be ruled out with 40 patients, regardless of the number of successes in the remaining patients. However, patients who have started their participation at this time should be able to complete the protocol.

#### Statistical analysis

Primary analysis of the primary endpoint: The exact two-sided 95% CI for the success rate will be computed from all patients with defined primary endpoint. In case the study will be stopped with n < 40, confidence limits will also be presented for the virtual scenarios with n = 40 and either success or non-success in all k “remaining” patients where n–k is the actual number of evaluable patients. The purpose of the primary analysis is to obtain advice for the hypothesis of a subsequent larger trial, as described in the sample size section.

For the clinical plausibility, we will examine if there might be differences in quality of life under the two stimulation modalities, and if these differences might be correlated with the measured functional data.

Note on the cross-over design: All analyses will be carried out just with methods for usual paired data. The effects of DBS are largely washed out after 48 h [12, 13] and our measurements will be obtained 4 weeks after a programming change. Therefore, carry over effects should be negligible. However, to rule out any wrong assumptions for the future trial, this study is being planned as a cross-over design, and the possibility of carry-over will be assessed for all endpoints under consideration.

The study database will be validated by the Clinical Trial Center Würzburg (CTCW). Web-based data entry during the study will ensure continuous data transfer to the CTCW. Central data checks and query management will ensure timely delivery and correctness of data. The data will be publically available after publication. ln accordance with the rules of good scientific practice and legal requirements, we will archive our data for at least 10 years.

### Eligibilty criteria

Inclusion criteria:

- Diagnosis of isolated dystonia (focal, segmental or generalized) treated by bilateral deep brain stimulation of the internal globus pallidus
- Sufficient time to complete clinical DBS programming after DBS surgery (> 6 months)
- Deep brain stimulation settings and dystonia medication stable for at least 3 months
- Understanding and agreement to the study and signed informed consent
  Exclusion criteria:
- Relevant comorbidities that might interfere with study endpoints (esp. palliative disease and severe neurologic or psychiatric comorbidities).

### Contacts

The study was planned by the Neurological Clinic of the University Hospital of Würzburg in conjunction with the Center for Clinical Studies Würzburg (CTCW) and the German Federal Ministry of Education and Research (BMBF). The study database will be validated by the CTCW. Web-based data entry during the study will ensure continuous data transfer to the CTCW. Central data checks and query management will ensure timely delivery and correctness of data.

### Perspective

DIPS is the first randomized, double-blind and controlled comparison for computer-assisted DBS programming for patients with dystonia. The purpose of this study protocol is to answer the question of which programming method is more effective in controlling the symptoms of dystonia. If superiority or equality of computer-based prediction of optimal stimulation parameters can be shown, this would have significant consequences for dystonia patients worldwide, as their programming is currently still predominantly dependent on the experience of the DBS programmer. In silico objective testing of all possible stimulation parameters within seconds would save patients lengthy programming sessions and sometimes months of programming titration.

## Data Availability

The datasets used and/or analysed during the current study will be available from the corresponding author on reasonable request.

## Abbreviations

BFMDRS: Burke–Fahn–Marsden Dystonia rating scale
CDQ24: Craniocervical dystonia questionnaire 24
DBS: Deep brain stimulation
DIPS: Dystonia Image-based Programming of Stimulation
SF-36: Short form-36
TWSTRS: Toronto Western Spasmodic Torticollis Rating Scale
VTA: Volumes of tissue activated
